# From Cereal Grains to Immunochemistry—What Role Have Antibodies Played in the History of the Home Pregnancy Test

**DOI:** 10.3390/antib12030056

**Published:** 2023-08-31

**Authors:** Kinga Lis

**Affiliations:** Department of Allergology, Clinical Immunology and Internal Medicine, Ludwik Rydygier Collegium Medicum in Bydgoszcz, Nicolaus Copernicus University in Toruń, ul. Ujejskiego 75, 85-168 Bydgoszcz, Poland; kinga.lis@cm.umk.pl

**Keywords:** antibodies, pregnancy test, Aschheim–Zondek, history, immunochemistry

## Abstract

Today, the home pregnancy test is the most frequently performed laboratory test for self-diagnosis (home diagnostic test). It is also the first laboratory test that has been adapted for self-use at home. This is probably because women have always wanted to know the answer to the question: “Am I pregnant or not?” and always preferred to know the answer to this question intimately and in a discreet way. The history of the pregnancy test is also an interesting example of how the discovery of antibodies and the development of in vitro diagnostic methods based on the antigen–antibody reaction were important for the development of laboratory and clinical diagnostics. Immunodiagnostic techniques (based on the antigen–antibody reaction) are currently the basis of modern specialist laboratory diagnostics, which is essential in clinical diagnosis. The history of the pregnancy test is an interesting one and dates back to ancient times. A pregnancy test is defined as a procedure intended to reveal the presence or absence of pregnancy. Nowadays, every pregnancy test is based on the detection of human chorionic gonadotropin (hCG) in urine or blood. Human chorionic gonadotropin is secreted by the placenta right after a fertilized egg cell implants in the uterus and can be detected in both the urine and blood of pregnant women. Urine pregnancy tests are convenient for self-use at home. Blood tests are performed in medical laboratories. Specialized laboratory methods not only detect hCG but also determine the concentration of this hormone. However, both of these methods are highly accurate and common. Throughout the ages, many different methods were used to detect pregnancy at the earliest stage. Grain, wine, and various small animals were used as research tools. These were both long-term and often unreliable; most were based on folk beliefs and superstitions. Animal pregnancy tests were the first biological tests used in this field. This was a significant advance in the accurate detection of relatively early pregnancy. Animal tests in modern times are considered cruel and inhumane, no matter how reliable their results can be. Their place is now taken by much more specific, more sensitive, and definitely more ethical immunochemical tests. The pregnancy test and the methods to find out whether a woman is pregnant have gone through massive transformations, from bioassays using plants to bioassays on animals to advanced immunochemical techniques and biosensors. Modern pregnancy tests are not invasive and are very sensitive. Nowadays, it takes only about 3 min to know the answer to the question: “Am I pregnant or not?”. However, it was not always as simple as it is today. This manuscript aims to show the important role played by antibodies in the development of laboratory and clinical diagnostics in the example of the interesting history of the pregnancy test.

## 1. Introduction

The discovery of antibodies played a significant role in the development of in vitro diagnostic methods. The knowledge of their properties and their skillful use became the basis for modern immunochemical techniques. Immunochemical techniques, on the other hand, have revolutionized laboratory diagnostics, which consequently translates into a wide clinical application [1]. An in vitro pregnancy test is defined as a procedure for determining the presence or absence of pregnancy based on in vitro diagnostic techniques [2]. The history of the pregnancy test is an example of the search for effective ways to diagnose various physiological and pathological conditions based on non-invasive diagnostic techniques. It leads from tests resulting from careful observation of the surrounding world, through simple diagnostic techniques based on organoleptic assessment and biological tests on animals, to techniques based on immunological reactions. The history of this test illustrates well how the antibody revolutionized in vitro diagnostics by introducing it to the essential canon of laboratory tests necessary in modern clinical medicine.

## 2. Modern In Vitro Pregnancy Test

Nowadays, all home pregnancy tests detect the presence of chorionic gonadotropin (beta (β) subunit) in the urine. Immunochemical techniques are commonly used for this purpose, primarily immunochromatography with visual, colmetric or biosensor detection. Although the first generation of these assays initially used polyclonal antibodies, monoclonal antibodies with high specificity for β-hCG are now commonly used. This strategy largely eliminates the possibility of false-positive results, which were caused by the high cross-reactivity of anti-β-hCG antibodies with other pituitary tropic hormones, especially human luteinizing hormone (hLH). This reactivity is related to the high structural similarity of some epitopes present in both molecules. This was problematic because high levels of hLH occurring at the time of ovulation contributed to false-positive pregnancy test results, which were a significant problem in tests based on polyclonal antibodies. The use of minoclonal antibodies has significantly improved both the sensitivity and specificity of home pregnancy tests. Chorionic gonadotropin is a pregnancy-specific hormone. In the blood of a pregnant woman, they appeared in detectable values about 6–8 days after the implantation of the embryo in the uterus. The highest concentration of this hormone is reached between the 7th and 10th week of pregnancy. The sensitivity of most pregnancy tests used today successfully detects β-hCG in the urine of a pregnant woman at a concentration of 25 U/L. This is the concentration that this hormone reaches in the urine of women even 3–4 days after implantation of the embryo in the wall of the uterus. This takes place before the expected menstrual bleeding. Therefore, it is possible to detect pregnancy even 7 days before the expected period (it is estimated that in 98% of cases, the result of the pregnancy test performed at this time will be positive). However, it is not recommended to perform this test so early, due to the relatively high possibility of spontaneous abortion in this early stage. All currently used home pregnancy tests are characterized by high sensitivity, specificity and simplicity of the technique. It is estimated that currently home pregnancy tests are the most frequently performed self-diagnosis test [2,3,4,5,6].

## 3. History of Pregnancy Test

For centuries, women have tried to recognize pregnancy in a reliable way as early as possible, although it was not always as easy as it is today. Women have always preferred to check it as individually as possible in intimate conditions and in the simplest and most reliable way possible. Although there were no scientific methods for detecting early pregnancy in a woman until the 1920s, and the first home pregnancy tests did not appear in the United States until 1976, the brilliant history of “laboratory” pregnancy tests began in ancient Egypt (Table 1) [7,8].

### 3.1. Ancient Egypt

The history of the pregnancy test dates back to ancient Egypt. Ancient Egyptian physicians have rightly noticed that the best material for laboratory tests to detect pregnancy is urine [8]. Four thousand years ago, the Egyptians developed the first in vitro diagnostic test to detect a unique substance present, according to their observations, only in the urine of pregnant women. It is the most famous and advanced of the ancient pregnancy tests. In Egyptian papyri dating from 1500–1300 B.C.E., there is a description of the pregnancy test used in ancient Egypt. This test consisted of watering cereal seeds (wheat and barley) with the urine of the examined woman every day for about 10 days. The sprouting of cereal grains meant that the woman was pregnant. No germination was a negative result (no pregnancy). According to the available descriptions, this test allowed not only to detect pregnancy but also to determine the sex of the developing child. According to the ancient Egyptians, if the germination of wheat preceded the germination of barley, it indicated a girl, and if the germination of barley came first, a boy was to be born [8,9,10,11,12]. It is worth noting, however, that there is no agreement in the translation of this papyrus either as to the cereal species used in the test or as to the interpretation of their germination in relation to gender recognition. Ghalioungui et al. [11], analyzing translations of the text made by various Egyptologists, note that three different grains are served (wheat, barley, and buckwheat). Also, the germination of wheat is not always considered an indicator of the female sex of the fetus since buckwheat is also mentioned as a cereal that indicates the female sex of the fetus. However, it can generally be assumed that the test itself, according to the available data, was performed as described above and that the sprouting of the cereals meant that the urine was from a pregnant woman.

The ancient Egyptian test of sprouting grains has been verified several times. In 1933, Manger [13] conducted an experiment on a sample of 100 pregnant women whose urine he used to water grains of wheat and barley. On the basis of the obtained results, he concluded that the urine of pregnant women actually accelerates the germination of cereals and that faster growth of barley than wheat means a girl, while neither accelerated nor delayed growth of barley means a boy. He estimated the effectiveness of the test in recognizing the sex of the child at 80%, although the conclusion was not consistent with the observations of the ancient Egyptians as to the relationship between the type of germinating grain and the sex of the fetus. In 1963, Ghalioungui et al. [11] performed an analogous experiment with 40 urine samples of pregnant women, using two types of control—urine samples of non-pregnant women and men, and distilled water. They found that 70% of the urine samples of pregnant women stimulated the germination of cereal grains. None of the urine samples of non-pregnant women or men showed such activity. Cereal germination was unrelated to fetal sex [11].

**Figure 1:**
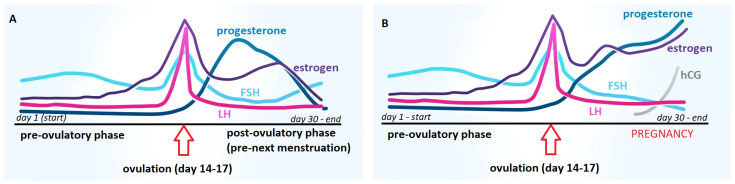
Comparison of the dynamics of hormonal changes during the menstrual cycle in women: (**A**) without conception and (**B**) with conception; FSH—follicle-stimulating hormone, LH—luteotropic hormone, hCG—human chorionic gonadotropin.

The ancient Egyptians established empirically that the urine of pregnant women could stimulate seed germination. This is probably due to the increased concentration of estrogen in the urine of women in the early stages of pregnancy (Figure 1). Human estrogens, like phytoestrogens, can affect the initiation of germination and stimulate plant development [14].

### 3.2. From Hippocrates to Gallen

In ancient Greece (ca. 400 B.C.), methods of detecting pregnancy, both the Hippocratic and Hellenic schools, were very similar to the methods used by the Egyptians. It was still believed that the urine of pregnant women contained life-giving components that stimulate seed germination [15,16]. However, methods directly interfering with the woman’s body were also readily used. One such method was the onion test. In this trial, an onion was inserted into the woman’s vagina and left there overnight. If, in the morning, a woman’s breath smelled of onions, it meant that she was not pregnant. It was believed that if the woman was pregnant, the smell of onion from the vagina could not get into the woman’s mouth. Pregnancy was also diagnosed when, after a woman consumed honey dissolved in water, her stomach was distended and painful. The Greeks, like the ancient Egyptians, also believed that if a woman felt nauseous after drinking milk or from the smell of beer, it meant that she was pregnant [10,16]. These theories, along with the development of trade, became known in all European countries. These tests were widely used until the Middle Ages.

### 3.3. From the Middle Ages through the Seventeenth Century

Perhaps slightly more empirical techniques were used in the Middle Ages. Using visual assessment of the physical characteristics of urine (e.g., color, clarity) to detect pregnancy became a popular method at the time. Doctors specializing in uroscopy, the so-called “Piss Prophate”, appeared in Europe; they specialized in the diagnosis of many diseases based on the visual assessment of a urine sample, i.e., uroscopy. Medieval uroscopy was a medical practice that involved the visual examination of urine for the presence of pus, blood, color translucence, or other lesions. The roots of uroscopy go back to ancient Egypt, Babylon, and India and were especially important in Byzantine medicine. These techniques were commonly used by Avicenna [16]. According to the guidelines of medieval uroscopy, the urine of a pregnant woman was clear, light lemon, and turning to whitish, with a foamy surface [10,16,17,18]. Other urine tests were also used in the Middle Ages. For example, it was believed that milk floated on the surface of a pregnant woman’s urine. At the time, some physicians believed that if a needle inserted into a vial of urine turned rust-red or black, the woman was probably pregnant [10,16,17,18]. Another popular test involved mixing wine with urine and observing the changes [18,19]. Today, we know that many of these tests used the presence of protein in the urine and the changes in urine pH of pregnant women due to hormonal changes associated with pregnancy. Indeed, protein-containing urine can be cloudy and frothy. Alcohol, on the other hand, reacts with some proteins in the urine, precipitating them. A more alkaline urine pH can darken some metals or remove rust. Pregnant women have higher levels of protein in their urine than non-pregnant women, and their urine pH is more alkaline, so these tests may have been quite effective for the time.

Various provocative pregnancy tests have also been used. Some doctors advised a woman suspected of being pregnant to drink a sweet drink before going to bed. If a woman complained of pain in the navel in the morning, the pregnancy was confirmed. In the 17th century, some doctors gave a woman a ribbon dipped in her urine to sniff. If the smell of this ribbon made the woman feel sick or vomit, it meant that she was probably pregnant [9,19]. In another test from the 17th century, a ribbon dipped in a woman’s urine was then burned in a candle flame. If the smell of smoke made the woman feel sick, it meant that she was probably pregnant [20]. It is difficult to find any other logical explanation for these tests than the natural tendency of pregnant women to feel excessively nauseous, which is due to hormonal changes caused by pregnancy.

### 3.4. Nineteenth Century

The nineteenth century did not bring anything new in this area; the main material for study was still urine. Nevertheless, researchers tried to approach this study in a more rational way. Attempts to link the microscopic examination of urine (bacteria or crystals) with pregnancy were made. In the 19th century, French doctors used a urine test called the “Kyesteine pellicle” as a method of pregnancy detection. The formation of a sticky film on the surface of the urine of pregnant women after standing in a vessel for several days was observed; it was called the early pregnancy membrane [21]. The diagnosis of pregnancy, however, was based mainly on the observation of physical changes in the body of a woman and the presence of characteristic symptoms of this condition, such as morning sickness [19].

### 3.5. From the 1920s to the 1960s

The first major steps towards constructing a reliable pregnancy test became possible in the 1920s after the discovery of a hormone present only in the urine of pregnant women. It was called human chorionic gonadotropin (hCG). That scientific discovery finally found a reliable, empirical marker that could be used for testing purposes. Since this discovery, all used pregnancy tests have been based on detecting the presence or absence of hCG in the urine [22,23].

Until the 1960s, pregnancy tests were mainly biological methods involving laboratory animals, mainly mice, rabbits, and a specific species of toads [21,22,23,24,25,26,27,28,29,30,31,32,33,34,35,36,37,38,39,40,41,42,43,44,45,46].

**Figure 2:**
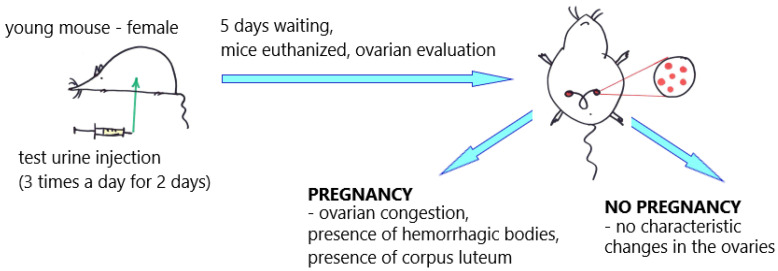
Aschheim–Zondek test (mouse); an illustrative scheme. The "red dots" illustrate changes in the ovaries of mice.

In 1927/1928, German scientists Selmar Aschheim and Bernhard Zondek developed the first biological pregnancy test, known as the Aschheim–Zondek test (A–Z test), which detects the presence of hCG in the urine (Figure 2). 

To test for pregnancy, the woman’s urine was injected into an immature male rat or female mouse. When the urine was from a pregnant woman, the injected rat exhibited an oestrous reaction despite its sexual immaturity. In the mouse version, sexually immature 3- to 4-week-old female mice weighing an average of 6–8 g were used for the A–Z tests. Five mice were used for each pregnancy test and injected subcutaneously with a total of 1.2–2.4 mL of urine in up to six doses over a period of forty-eight hours. The result was read, on average, after one hundred hours, and a positive result (pregnancy) was indicated by ovarian congestion, the presence of hemorrhagic bodies and numerous corpora lutea. Changes in the ovaries were assessed microscopically. Had the patient not been pregnant (negative), there would have been no such reaction in the mouse ovaries. The positive result A–Z test in the urine of pregnant patients was associated with the presence of gonadotropin in the urine, which is associated with pregnancy. It is also interesting that during the early A–Z test studies, scientists discovered that testicular tumors can also produce hCG [22,24,28]. Furthermore, hyperemia of the rat ovary has been reported to be present after the administration of luteinizing and luteotropic gonadotropins but not after the use of the follicle-stimulating hormone [29].

**Figure 3:**
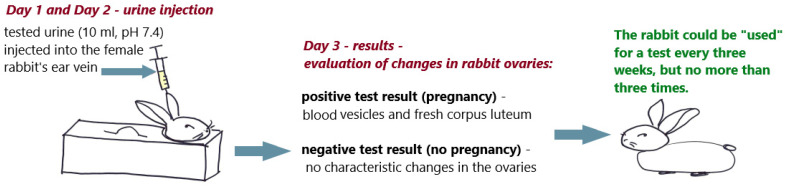
Friedman test (rabbit); an illustrative scheme.

Then, rats and mice were replaced by rabbits. In the early 1930s, Maurice Harold Friedman, a physician and physiology researcher at the University of Pennsylvania, developed the “rabbit test” (Friedman test) as a modification of the A–Z test [22,26]. Friedman proved that a single dose of urine from a pregnant woman was able to induce ovulation in sexually immature female rabbits. Importantly, the test did not require killing the rabbit, which was necessary when tested on mice [17,18,19]. The technique of the Friedman test was that the female urine was injected intravenously into the female rabbit (into the ear vein) (Figure 3). If hCG was present, the female rabbit ovulated within 48 h. Evaluation of the ovaries required the opening of the rabbit’s abdomen, which was performed under anesthesia without killing the animal. Blood follicles and fresh corpora lutea were considered positive [30,31,32,33].

The rabbit could be reused for the test after about three weeks, but not more than three times. In practice, however, most rabbits that tested positive were killed after the test [30,31,32,33]. In the days when pregnancy tests were done on rabbits, the colloquial term “rabbit died” was synonymous with “you are pregnant”, which can be found in humorous phrases used to this day.

Interestingly, the accuracy of the A–Z test and the Friedman test was estimated to be around 82.5–99.5%. Both of these tests were used on a massive scale from the 1930s until the early 1960s [34,35,36,37,38].

**Figure 4:**
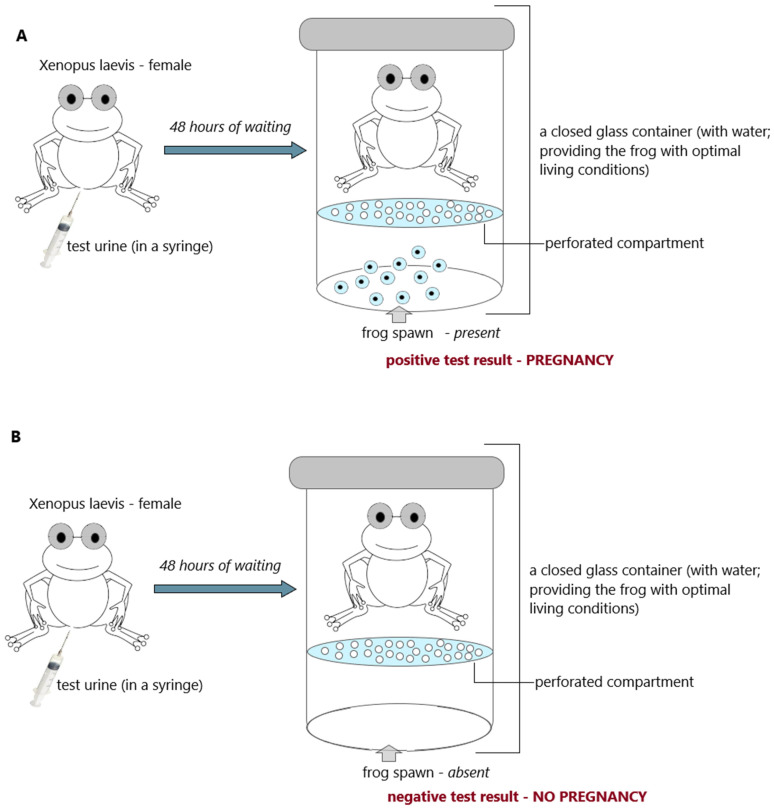
Hogben test (frog; *Xenopuslaevis*); (**A**) positive result (pregnancy) and (**B**) negative result (no pregnancy); an illustrative scheme.

Mice, rats, and rabbits were replaced by frogs. The frog pregnancy test (“frog test”) was developed by the British zoologist Lancelot Hogben. Hogben’s test involved injecting a woman’s urine into an African clawed frog (*Xenopus laevis*). It is the only species of toad whose females are sensitive to gonadotropins present in the human urine of pregnant women. If the woman was pregnant, the frog ovulated within 2 to 8 h (Figure 4A). However, if the urine was from a non-pregnant woman, the frog did not spawn (Figure 4B) [26,39,40,41,42].

In 1947, Carlos Galli Mainini developed a pregnancy test using the male toad *Bufoarenarum Hensel.* This toad produced sperm in response to stimulation with chorionic gonadotropin from the urine of a pregnant woman, which was injected into the lymphatic sac of a male toad. The test result was available after three hours, and the assessment was minimally invasive for the animal. Sperm were viewed in urine collected from the toad’s cloaca [43,44]. This test has also been adapted to various other species of locally occurring frogs and toads [45,46,47].

The frog/toad tests had two significant advantages over tests using mice, rats, or rabbits. Most importantly, the frogs could be used for testing many times, and reading the results did not require either killing or opening the animal’s abdominal cavity because the frog’s eggs are secreted to the outside. The second significant advantage of this test was the relatively short waiting time for the result compared to earlier tests using different mammals. The Hogben frog test was the world standard for laboratory pregnancy detection for decades [39,40,41,42].

Interestingly, when biological tests were replaced by immunochemical tests in the 1960s, some hospitals released unwanted African clawed frogs (*Xenopus laevis*) imported from South Africa into the wild. According to a study by Vredenburg et al. [47], at that time, a deadly fungus (*Batrachochytrium dendrobatidis*) was found in frogs imported from South Africa, which decimated the native American frogs, causing an ecological disaster.

All of these animal tests worked because a pregnant woman’s urine contains a pregnancy-specific hormone, human chorionic gonadotropin (hCG). hCG is made by a woman soon after conception and plays a critical role in the implantation of the embryo into the uterus. However, these bioassays were expensive, sometimes required the killing of animals, and the waiting time for test results was several days. Unfortunately, these tests were also at risk of false positives, especially when tested on female small mammals, due to the similarity between hCG and luteinizing hormone (LH). Most of these bioassays were, in fact, unable to distinguish between the two hormones, except when hCG levels were extremely high [24,42,48].

### 3.6. 1960s—Agglutination Tests

Animal bioassays (mice, rats, rabbits, and frogs) were the only practical way to detect pregnancy for four decades. The year 1960 began the era of immunological tests, which also found application in the detection of early pregnancy, allowing the abandonment of animal testing and enabling the development of the technology for rapid, sensitive, and specific home tests [24]. The ability to produce polyclonal antibodies (by immunization of animals) and use them as diagnostic reagents has generally revolutionized laboratory diagnostics and opened up a wide range of possibilities [49].

**Figure 5:**
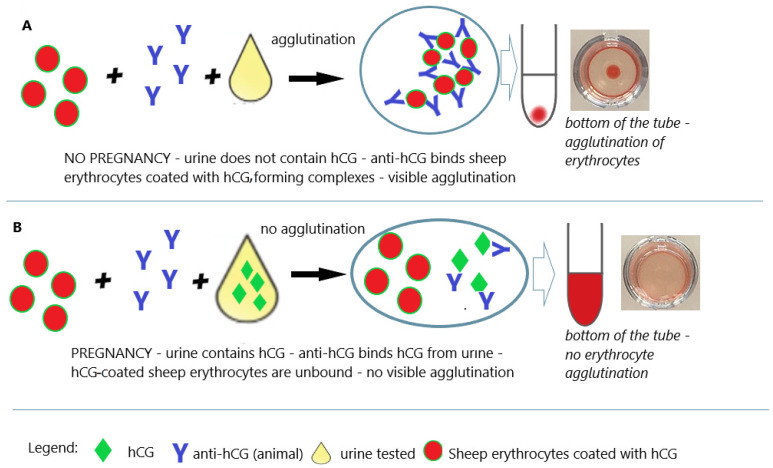
Pregnancy test based on the hemagglutination inhibition test. (**A**) Negative result (no pregnancy); (**B**) positive result (pregnancy); an illustrative scheme.

In the early 1960s, Wide and Gemzell announced the Wide–Gemzell test as an immunological hemagglutination inhibition method for diagnosing pregnancy [50]. The test using the agglutination inhibition test was the first pregnancy test that successfully opened the way for tests for home use [50]. These tests were based on agglutination inhibition reactions of chorionic gonadotropin-coated sheep blood cells (Figure 5). Since cells were used in the testing process, this test was an immunoassay rather than a bioassay.

This test method detected hCG concentrations of 200 to 300 IU/L, and the analysis time was only 90 min. The test used a suspension of hCG-sensitized sheep erythrocytes preserved in formalin and rabbit anti-hCG antibodies. A suspension of sensitized sheep erythrocytes is combined with test urine and rabbit anti-hCG antibodies. If the test urine does not contain hCG (urine of a non-pregnant woman), anti-hCG causes agglutination of sensitized sheep erythrocytes (Figure 5A). If the test urine contains hCG (urine of a pregnant woman), anti-hCG antibodies bind to hCG from the urine and do not agglutinate sensitized sheep erythrocytes (Figure 5B) [50,51]. While this test was much faster and less expensive than conventional bioassays, it was relatively insensitive, especially for early pregnancy diagnosis, due to cross-reactivity with various drugs. Problems of this kind have been reported to require careful reading of results, as the presence of certain substances in the urine may result in false-negative or false-positive results. The predictive value (positive and negative) of hemagglutination pregnancy tests was estimated at an even 98%, although not all commercial blood cell tests available at that time were equally accurate [51,52]. Urine hemagglutination tests have also been an effective tool for detecting ectopic pregnancy [53].

**Figure 6:**
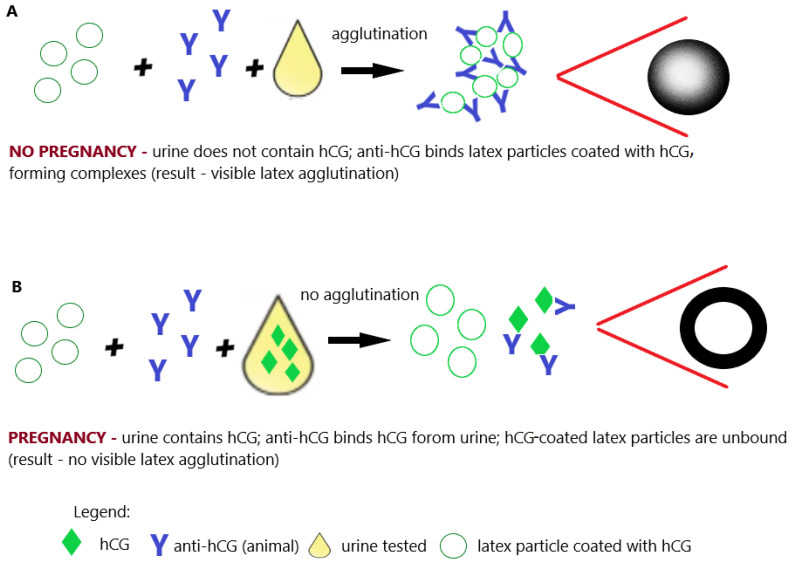
Pregnancy test based on the latex agglutination inhibition (slide) test. (**A**) Negative result (no pregnancy); (**B**) positive result (pregnancy); illustrative scheme.

Almost simultaneously with hemagglutination tests, slide(latex) agglutination tests began to be used, in which sheep blood cells were replaced with latex particles, and the agglutination reaction was performed on glass or plastic (preferably black) plates (Figure 6A,B). For this reason, these tests are sometimes also called latex agglutination tests [54,55,56]. Nevertheless, hemagglutination tests and slide (latex) agglutination tests turned out to be at least as reliable in detecting pregnancy as biological tests, which were common at the time, and successfully replaced them [51,54,55,56].

### 3.7. 1970s—Time of the Radioimmunoassay

In the 1970s, the structure of the hCG molecule was precisely determined. This allowed the construction of immunological tests unequivocally distinguishing hCG from luteinizing hormone (LH) [57,58,59,60]. Further research (1980s/1990s) into the effects of hormones on reproduction led to the development of a way to identify and measure hCG [61,62]. This knowledge, along with the development of immunology and immunochemistry, has become the basis for the construction of specific pregnancy tests that can clearly detect pregnancy in its early stages.

In 1966, Midgley first measured hCG and luteinizing hormone using a radioimmunoassay that ranged from 25 IU/L to 5000 IU/L [63]. Early RIA techniques were unable to distinguish between LH and hCG due to their cross-reactivity with specific antibodies. In order to overcome the cross-reaction between LH and hCG, the assay was performed at a concentration that did not interfere with LH, but the sensitivity of hCG deteriorated as a result.

**Figure 7:**
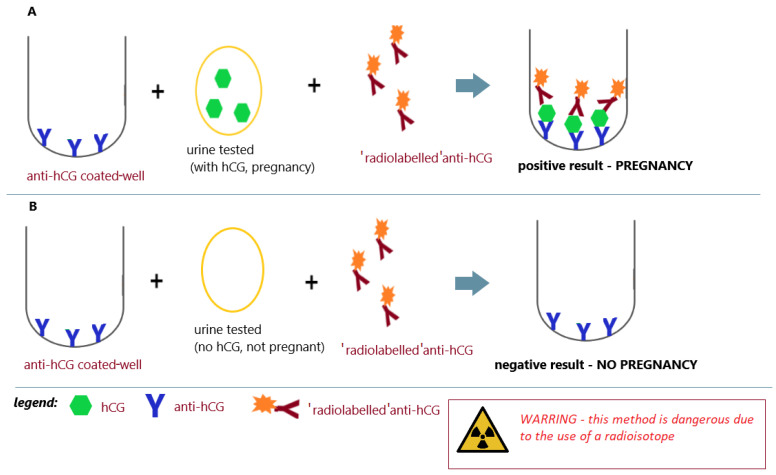
Radioimmunoassay for pregnancy; (**A**) positive result (pregnancy) and (**B**) negative result (no pregnancy); an illustrative scheme.

In 1972, Vaitukaitis et al. [64] published their paper describing the hCG beta-subunit radioimmunoassay that could finally distinguish between hCG and LH, therefore making it potentially useful as an early test for pregnancy. Antiserum directed against the beta-subunit of human chorionic gonadotropin (β-hCG) was used in the assay. With this strategy, it was possible to selectively measure hCG in samples containing both human pituitary luteinizing hormone (hLH) and hCG. The high levels of hLH observed in samples taken during the luteinizing phase of the menstrual cycle or from castrated patients had no effect on the specific detection of β-hCG by this radioimmunoassay (Figure 7A,B) [64].

The sensitivity and specificity of this test have been validated by other researchers. The test has been found to be of particular value in those conditions where hCG concentrations, as measured by previously described assays, will be affected by circulating hLH levels, i.e., early implantation, unruptured ectopic pregnancy, threatened abortion, and trophoblastic disease during chemotherapy [65,66]. The RIAs for the detection of the beta-subunit of chorionic gonadotropin performed well in urine, plasma, and serum [67,68]. Clinical specificity has been estimated at 99%, and clinical sensitivity in the range of 89–96% for serum measurements [68]. In 1974, Saxena et al. [69] developed a radio receptor test (RRA) for determining the beta-subunit of hCG. The sensitivity of this test was 5 IU/L, which made it possible to detect pregnancy already on the 6th–8th day of its duration, and the analysis time was reduced to 1 h. It is worth noting that a significant increase in the sensitivity and specificity of pregnancy tests was made possible only in the 1980s by the introduction of monoclonal antibodies for the hCG beta-subunit instead of the previously used polyclonal antibodies [70].

From a technical point of view, it is very important that the tests developed by Vaitukaitis et al. [64] or Saxena et al. [69] were based on the detection of the intensity of ionizing radiation. Radioimmunoassays (RIAs), radioimmunometric assays (IRMAs), or radioreceptor assays (RRAs) are immunoassays that use radioactively labeled molecules to detect immune complexes formed during the antigen/antibody reaction [71,72]. The era of RIA testing in laboratory diagnostics began in 1959. The first immunological technique was the isotopic dilution radioimmunoassay developed by Rosalyn Yalow and Solomon Berson to detect insulin in human blood. For this achievement, Yalow was awarded the Nobel Prize in Physiology or Medicine in 1977. In the 1960s and 1970s, the RIA became an important tool in biological research [72]. Radioimmunoassays using antibodies labeled with radioisotopes have revolutionized laboratory diagnostics and enabled the detection of various biological substances (including, for example, hormones), even at very low concentrations (Figure 7). Unfortunately, as research that was dangerous and required special conditions protecting against radiation, they could not leave the laboratory; it was definitely not a home testing strategy.

**Figure 8:**
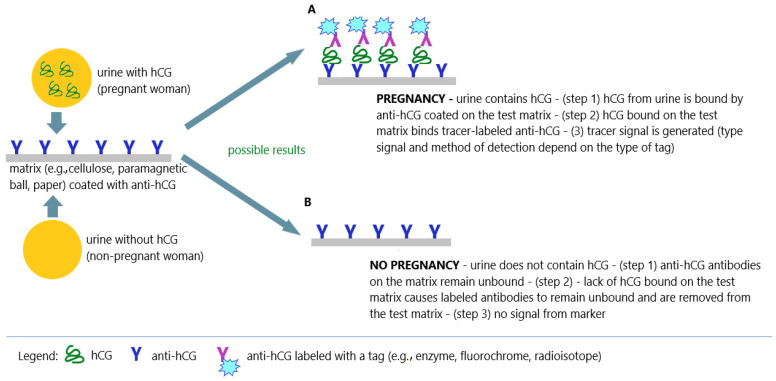
Immunochemical techniques in pregnancy tests; (**A**) positive result (pregnancy); (**B**) negative result (no pregnancy); an illustrative scheme.

The beginnings of immunochemical techniques based on the use of antibodies labeled with enzymes date back to the early 1970s [73]. However, it was not until the 1980s that durable and stable immunological reagents were obtained that could be successfully used in standard laboratory techniques [74]. These techniques revolutionized the entire area of immunological diagnostics and gradually replaced radioimmunoassays. Currently, most immunochemical measurement techniques use sandwich and competitive immunoenzymatic methods with colorimetric or luminescence detection (Figure 8).

At that time, sensitive and specific immunochemical techniques were also developed, based on the standard ELISA (Enzyme-linked Immunosorbent Assay), using anti-β-hCG monoclonal antibodies. The lower limit of detection was 0.2 μg/L after a 2 h incubation with serum sample at room temperature and a 30 min incubation with enzyme substrate at room temperature after washing away excess enzyme conjugate. Within-assay precision was less than 6% [75].

### 3.8. From 1970 to Today—Timeof the Home Pregnancy Tests

The home pregnancy tests were the first tests intended for self-administration at home. These tests contributed greatly to a kind of social and moral revolution. On the one hand, the proponents of these tests pointed to the benefits of using them. These tests gave women the opportunity to detect pregnancy early, which in turn allowed for appropriate preventive measures to ensure optimal conditions for fetal development from the early stages of pregnancy. This increased the chances of carrying the pregnancy to term and giving birth to a healthy child. On the other hand, these tests also had many opponents. They were seen as feminist tools to allow women to control births. Opponents of home pregnancy tests indicated that early and independent detection of pregnancy gave women the opportunity to perform abortions and increased women’s sexual freedom [22,76,77].

The basis of modern home pregnancy tests are immunochemical techniques and, based on these, immunochromatographic techniques. Although there were already rapid immunochemical tests [77,78], the first commercially available home pregnancy tests were not constructed using these technologies but used less advanced immunological techniques, such as the complement fixation test.

The first home pregnancy test was approved by the FDA (Food and Drug Administration) in the United States in 1976. This was the “e-p-t” (Early Pregnancy Test), later known as the “Error Proof Test (e-p-t)”. The e-p-t test became the first commercially available home pregnancy test (in the USA) and cost about $10. Around the same time, three other tests were also approved that were considered “essentially equivalent” to e-p-t, i.e., Predictor, ACU-TEST, and Answer [9,77,79,80,81]. Although it is worth noting that the Predictor test was patented as early as 1968, the FDA delayed its recognition, and initially, from 1970, it was available only in Canada [80].

The first home pregnancy tests included test tubes, a rack, droppers, dried sheep blood cell capsules, and anti-hCG-positive serum. The procedure for performing the test required careful execution of a complex, 10-step process. The time required to complete the entire test was 2 h. The tubes had to be positioned in a vibration-free location throughout the procedure. The accuracy of the test was 97% for a positive result and 80% for a negative result [77,80,82,83].

**Figure 9:**
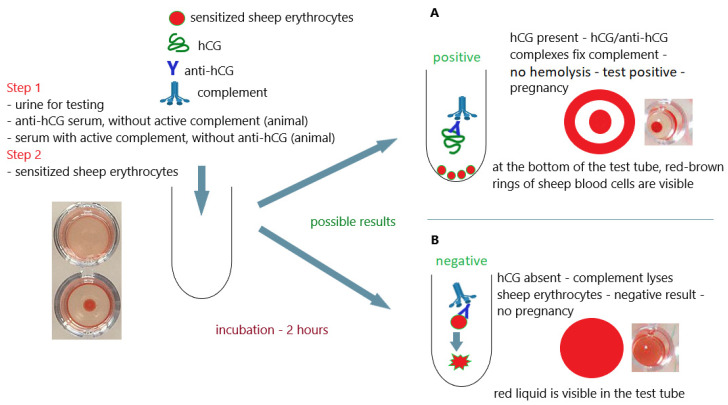
Complement fixation test (complement fixation inhibition test) home pregnancy test; (**A**) positive result (pregnancy); (**B**) negative result (no pregnancy); an illustrative scheme.

All pregnancy tests available at that time used the immunological reaction based on the complement fixation test as a method of detecting hCG (Figure 9A,B).

The complement fixation test is an immunological technique that allows the detection and determination of an antigen or antibody in the test material (e.g., urine, serum). If antigens are detected in this test, it is also necessary to use serum-containing antibodies specific for the detected antigens. This is basically referred to as a complement fixation inhibition test. Hemolysis of sensitized sheep erythrocytes caused by activation of complement proteins is used to read the test result. The complement system is a group of about 30 proteins present in the serum of vertebrates that mutually regulate their own activity (proteolytically activate, stabilize, or inhibit). Cascade activation of complement proteins is caused by antigen–antibody complexes (immune complexes). If the activating immune complexes are localized on the surface of sheep erythrocytes, this causes hemolysis of those cells. Activation of the proteins of the complement system leads to the formation of the membrane attack complex (MAC)—a complex of complement proteins—with components C5bC6C7C8(C9)n, which has the ability to incorporate into the cytoplasmic membrane (sheep erythrocyte membrane) and form a channel in it (this causes cell lysis). Under physiological conditions and various pathological processes, the MAC complex can attack the cell membranes of various eukaryotic cells, including the body’s own cells and the membranes of bacterial cells (cell membrane and outer membrane of Gram-negative bacteria). It is one of the main elements of the body’s immunity [84,85,86].

Complement fixation pregnancy tests used hCG-coated sheep cells on the surface, animal serum-containing anti-hCG antibodies, and rabbit serum as a source of complement proteins. In order to perform the test, the woman’s urine and the previously mentioned ingredients were added to the test tube. If the test urine did not contain hCG (no pregnancy), anti-hCG antibodies bound to hCG on the surface of the sheep erythrocytes, which activated the complement protein and led to lysis of the erythrocytes (Figure 9B). If the test urine contained hCG (pregnancy), the anti-hCG antibodies were bound by the hCG in the test urine and could not bind to the hCG on the surface of the sheep red blood cells. In this situation, complement was not activated on the erythrocyte membrane and did not cause their lysis (Figure 9A). Therefore, the lack of hemolysis indicated a positive test result and the presence of pregnancy in the examined woman. The ongoing process of hemolysis was, in turn, a confirmation that the tested hCG antigen was absent in the test sample, which proved the absence of pregnancy [26,80,83].

The complement fixation test was previously successfully used in laboratory conditions to perform pregnancy tests [87,88,89], but the observation of the results, their evaluation, and their correct interpretation caused considerable difficulties for people who were not professionally involved in performing this type of procedure. An interesting solution for reading the results was proposed by Margaret Crane when she designed the test set, which then appeared on the market under the name Predictor. In this solution, the result was evaluated as the reflection of the bottom of the test tube in the mirror mounted under it, and the whole set was contained in one plastic box. This assay also used complement fixation reactions [80]. This solution greatly facilitated the observation of the test results at the bottom of the tube. This is particularly important when you consider that home tests are performed and interpreted by people who do not have any special preparation and experience for this.

Between the 1980s and 1990s, rapid advances in laboratory diagnosis were made, which is also true for pregnancy tests. In the 1980s, the execution time of a home pregnancy test was substantially reduced [90]. In 1982, it was one hour (Daisy 2 pregnancy test kit: August 1982), while in 1985, it was only 20 min (First Response: November 1985). In 1982, it was one hour, while in 1985, it was only 20 min. In 1986, the waiting time for the pregnancy test was no longer than 10 min (“The pregnancy test can now be used 1 day after a missed period, and results display in about 10 min. e.p.t^®^ gives women the peace of mind that comes with knowing sooner”, 1986) [90].

**Figure 10:**
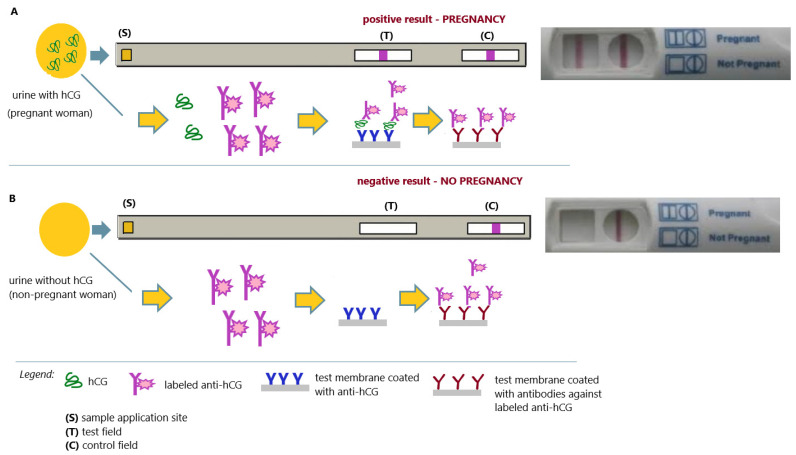
Immunochromatographic pregnancy test; (**A**) positive result (pregnancy); (**B**) negative result (no pregnancy); an illustrative scheme.

At the turn of the 1980s and 1990s, there was dynamic development of immunochemical methods in which radioactive tracers were successfully replaced with enzymatic tags. In addition, the technique for obtaining specific monoclonal antibodies was improved. This allowed the development of simple, sensitive, specific, and safe immunochemical methods that were successfully used in home pregnancy tests. Immunochromatographic pregnancy tests have come into widespread use (Figure 10A,B).

An immunochromatographic test (ICA), also called a lateral flow test, is a simple tool used to detect the presence or absence of a target analyte (usually an antigen or antibody). Immunochromatography is a laboratory technique that combines chromatography (separation of sample components based on changes in the way they pass through a specific sorbent) with immunochemical reactions (antigen–antibody reactions using a detection label). The most commonly used immunochromatographic system is a test strip (usually a cellulose membrane). Depending on the target analyte, antigens (for antibody detection) or antibodies (for antigen detection) are immobilized on a nitrocellulose membrane (test solid phase) in a defined area of this membrane (test result reading field—T). In the assay control area (control reading field—C), antibodies specific to the class and type of detection antibody (labeled with a chromogenic marker) are immobilized. In the space of the membrane between the sample application site and the T-field, free antibodies specific for the target analyte, labeled with a chromogen, are applied. The process begins when the test sample is added to the membrane (application site—S). The liquid matrix of the test sample (urine, serum, plasma, specific buffer) constitutes the liquid phase of the test. As the procedure progresses, the sample moves along the test membrane. In the area between the sample application area and the T-field, the analyte combines with specific chromogen-labeled antibodies (added in excess) to form immune complexes (analyte/antibody; the antibody can be specific for the analyte (when antigens are detected) or for solid fragments that determine the class of antibodies (when antibodies are detected)). Excess labeled antibodies remain unbound. In the course of the process, the resulting immune complexes (analyte/labeled antibody) are captured by specific antibodies (antigen determination) or antigens (antibody determination) immobilized in the T-field (giving a color line in this area). Excess unbound labeled antibodies are bound in region C (giving a control line). In the absence of the target analyte (antigen or antibody) in the test sample, immunological complexes are not formed in the initial area of the membrane, which means that the labeled antibody does not bind in the T-field, and no color line is formed. In this situation, only a control line is created where labeled antibodies are bound. The absence of a control line indicates incorrect performance of the test or some other (unspecified) reason for which the test is defective. In this situation, the test result is non-diagnostic, and the presence or absence of a colored line in the T-field is irrelevant [91]. The classic example of an immunochromatographic test is the home pregnancy test (Figure 10) [92].

In 1987, e.p.t^®^ introduced a new strip technology that eliminated the “chemistry kit” to replace the old home pregnancy tests. These tests still detected the presence of hCG in the urine, but the test kits were no longer like a “chemistry kit”, and the test took a maximum of 5 min to complete. The year 1996 marked the beginning of the currently used strip and cassette home pregnancy tests [42,90].

Nowadays, there is an effort to standardize the home pregnancy tests available on the market. First of all, attention is drawn to the sensitivity of the test, i.e., the minimum concentration of hCG at which the test should give a positive result in >95% of cases and the so-called pregnancy detection rate when the test is performed before menstruation, i.e., the percentage of detected pregnancy results on the day in relation to the day of expected menstruation. It is also important that the results obtained by tests from different manufacturers are comparable [5,93].

Modern home pregnancy tests are up to 99 percent accurate, independent of the presence of certain medications [7]. According to estimates by Cole et al. [94], the sensitivity of the test at the level of 12.5 mIU/mL is needed to detect 95% of pregnancies on the day of expected menstruation. In turn, according to a study by Gnoth and Johnson [5], a test detecting 25 mIU/mL hCG (which is the second percentile of hCG concentration on the day of expected menstruation) has a 99% pregnancy detection rate. It seems necessary that the minimum sensitivity of pregnancy tests available for self-administration at home should not be lower than 25 mIU/mL hCG. In fact, according to research by Tomlinson et al. [95], the sensitivity of home pregnancy tests from different manufacturers, and thus the ability of these tests to detect pregnancy at its early stage and the clinical value of the result, can vary greatly. Among the tests evaluated by these researchers, minimal hCG levels of 25 mIU/mL were detected in only 8.3–87.5% of urine samples from women in early pregnancy that should have already been detected by home pregnancy tests. Johnson et al. [96], based on their comparative studies of various commercially available pregnancy tests, point out that many home pregnancy tests commonly used by women are not as accurate as claimed on the packaging. This can cause false-negative results in the early stages of pregnancy, with important clinical implications.

It is interesting that some manufacturers of currently available home pregnancy tests declare that these tests can also be performed at an earlier stage of pregnancy, up to 6 days before the expected period, obtaining reliable results [97,98] (with a sensitivity of only 71%) [97]. Nevertheless, due to both the low sensitivity of pregnancy tests in such early stages of pregnancy and the possibility of spontaneous miscarriage in very early stages (from undetermined natural reasons), it is not recommended to perform home pregnancy tests before the day of expected menstruation [99,100].

### 3.9. The Twenty-First Century—The Timeof Digital Pregnancy Tests

**Figure 11:**
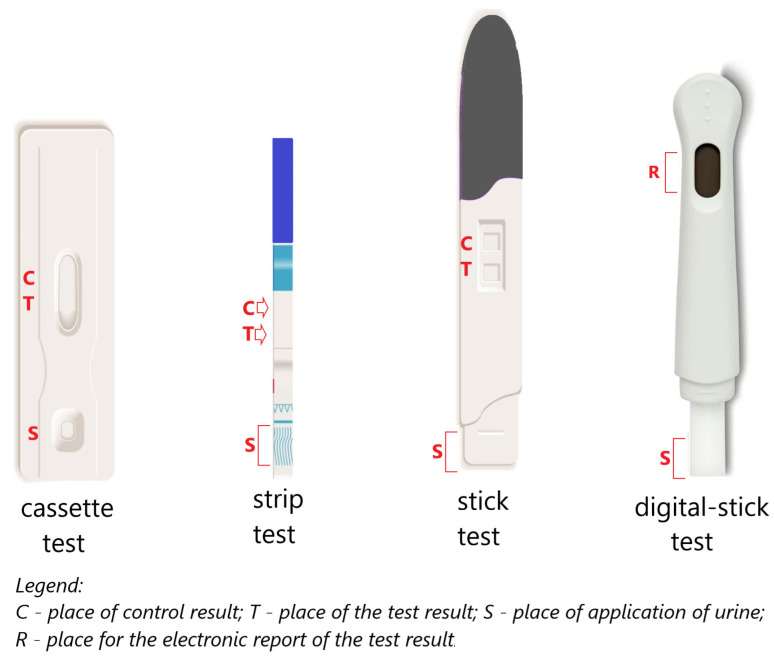
Examples of types of home pregnancy tests.

Currently, home pregnancy tests are available in various forms (Figure 11). The form of the test does not affect its quality and sensitivity; it only affects the convenience of its use.

Electronic pregnancy tests appeared at the beginning of the 21st century [17] as a response to the difficulties associated with the correct interpretation of results showing in the form of colored lines. Reading traditional immunochromatographic tests may cause some difficulties when the appearing color lines are very poorly stained [101].

**Figure 12:**
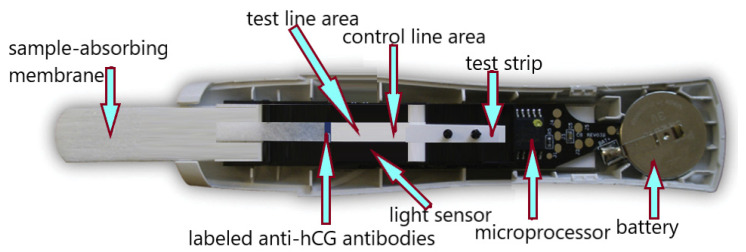
Home digital pregnancy test—construction scheme based on Clearblue Easy Digital Pregnancy Test (SPD Swiss Precision Diagnostics GmbH).

The digital (electronic) pregnancy test was introduced in 2003. The modified digital Clearblue Easy consists of a test chip in an absorbent tester and electronic and optical components in molded housings. The device comes in a ready-to-use format and no longer requires assembly before use. The test uses an immunochromatographic technique with biosensor reading. The digital test uses the same type of antibody as the original Clearblue test kit from which it was constructed (i.e., anti-beta hCG, goat anti-rabbit, anti-alpha hCG, and rabbit IgG). The Clearblue Easy Digital Pregnancy Test (SPD Swiss Precision Diagnostics GmbH) is a sandwich immunoassay that uses monoclonal antibodies specific for hCG and uses chromatography principles to separate bound and free markers. A reader is incorporated into the test stick that measures the reflectance of the light falling on the test and control lines to determine the test result or whether an error has occurred. The measurement result is converted into an electronic signal. The display shows whether you are pregnant (“pregnant”) or not (“not pregnant”) (Figure 12) [102]. 

**Figure 13:**
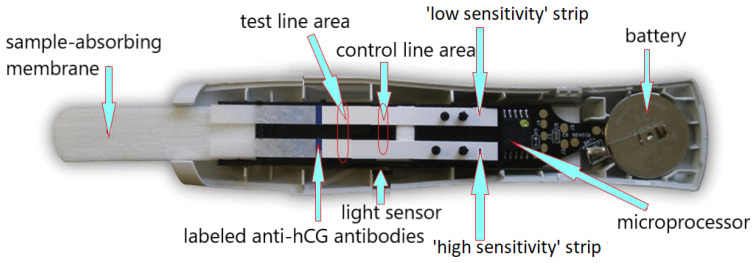
Home digital pregnancy test with weeks estimation—construction scheme based on Clearblue Advanced Pregnancy Test with Weeks Estimator (SPD Swiss Precision Diagnostics GmbH).

A digital test with an estimated gestational age, Clearblue Advanced Pregnancy Test with Weeks Estimator (SPD Swiss Precision Diagnostics GmbH), is also available. The technology used in it is also based on an immunochromatographic reaction with biosensor detection, and the time elapsed since ovulation is estimated on the basis of the intensity of the color reaction, which generates an adequate light signal. The test consists of two membranes calibrated for lower and higher sensitivity (Figure 13) [103,104]. 

The Clearblue Advanced Pregnancy Test with Weeks Estimator (SPD Swiss Precision Diagnostics GmbH) categorizes the gestational age in three ranges:1–2 weeks, 2–3 weeks, and >3 weeks [105]. According to research by Johnson et al. [104], the test is highly consistent with the gestational age estimated on the basis of the assessment of the day of ovulation preceding conception and the gestational age assessed by ultrasound. However, it is worth noting that the test does not estimate the age of pregnancy as it is commonly accepted (based on the day of the last menstruation) but from the day of ovulation. This may be the cause of inconsistency and incorrect estimation of gestational age by self-testing women [106].

Clearblue home digital pregnancy tests are not the only commercially available tests of this type. Among other electronic tests, an interesting solution is a version that includes an application (by Bluetooth) that helps both to carry out the test and contains a lot of instructions and advice for the tester, helpful both in the case of a positive (“yes+”) and negative (“no−”) result (FIRST RESPONSE™ Digital Pregnancy Test; Church & Dwight Co., Princeton, NJ, USA) [107].

The main advantage of electronic pregnancy tests is that they make it easier to read the result. In the result window, there are no colored lines but clear information, such as “pregnant” (for a positive result) or “not pregnant” (for a negative result). This form of reporting the result definitely facilitates its interpretation. This is extremely important if you take into account that home pregnancy tests are performed, in the vast majority, by people who are not professionals in laboratory analysis. 

An interesting fact is that digital home pregnancy tests raise a lot of controversy with regard to the environment and climate protection. Organizations draw attention to the fact that the disposal of electronic components and batteries contained in these tests is difficult, and these tests contribute to increased pollution and the amount of non-degradable and toxic waste [108].

## 4. Summary and Conclusions

The home pregnancy test is probably the most widely used self-diagnostic test [109,110]. Methods of detecting pregnancy at the earliest possible stage of its duration have been the subject of intensive search since ancient times. In ancient times, observations from the surrounding world were used, such as the fact that the urine of pregnant women stimulates seed germination. As we know today, it owes these properties to the gonadotropins contained in it, which activate the processes of plant germination. These were the first simple biological methods. In addition to biological methods, ancient physicians also used observations of the changing body of a pregnant woman. Later, the search for the perfect diagnostic method for detecting pregnancy continued to focus on urine. Initially, attempts were made to use its properties resulting from chemical changes associated with pregnancy, such as the ability to remove rust from metals or foaming or precipitating sediment from wine. Today, it is known that these properties result from changing pH or increased protein concentration, which is observed in the urine of pregnant women. Medieval times did not bring any new discoveries in this field. The real breakthrough in the area of pregnancy detection was the discovery and description, in the 1920s, of the endocrine system, hormones, and, above all, chorionic gonadotropin (CG), which is a pregnancy-specific hormone. These discoveries were followed by more pregnancy bioassays using a variety of laboratory animals (like mice, rats, rabbits, and frogs). These tests were quite sensitive and specific. Unfortunately, their biggest drawback was the huge number of animals that were killed during testing. The discovery of the possibility of producing specific polyclonal and then monoclonal antibodies, as well as the techniques of conjugating antibodies with various substrates and labels, opened the era of immunoassays. The development of immunological methods meant that it was no longer necessary to kill animals to detect pregnancy in a woman. In turn, the precise molecular characteristics of the chorionic gonadotropin molecule increased the specificity of pregnancy tests and freed them from the interference of other tropic hormones, especially luteotropin (LH). This significantly increased the specificity of these tests and improved their sensitivity. Radioimmunoassays have contributed to an incredible increase in the sensitivity of pregnancy tests. Unfortunately, their disadvantage was that, as dangerous methods, they could not be used outside the laboratory. Only the introduction of chromogenic and enzyme markers made it possible to construct pregnancy tests in the form that is still used today. Currently, home pregnancy tests are based on the technique of immunochromatography. They use polyclonal or monoclonal anti-hCG antibodies and chromogenic tags. They have the form of cassettes, strips, or sticks. The reading is either visual (test and control lines) or electronic (pregnancy or non-pregnancy appears on the test display). Regardless of the stage of advancement, home pregnancy tests detect the presence of hCG in the urine of pregnant women.

**Figure 14:**
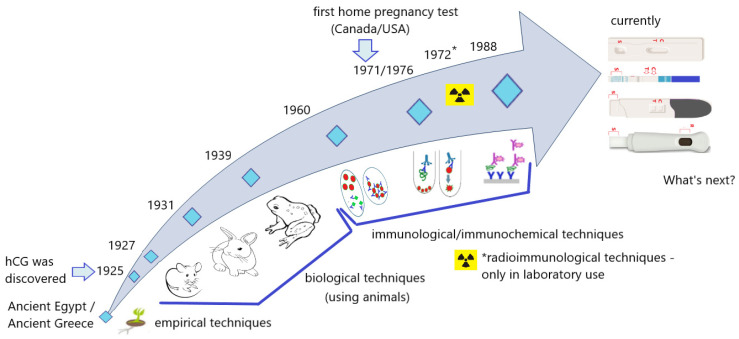
Home pregnancy test timeline.

In conclusion, home pregnancy tests have come a long way, from the first grain of cereal soaked in a woman’s urine to the immunochromatographic pregnancy tests most women will probably do at least once in their lives. They have evolved from primitive urine bioassays to simple-to-use and precise products based on sensitive and specific immunological methods. They have evolved from doctor’s offices and laboratories to households, from plants to animal tests, to quick tests that answer the important, very intimate question—“Am I pregnant or not?”, and from several days to several minutes of waiting for a response (Figure 14).

It must be emphasized that the ancient Egyptians were good observers of nature and had some luck using urine as a test material to detect pregnancy, but antibodies revolutionized this test and introduced it to women’s homes, probably forever.

## Figures and Tables

**Table 1:** History of pregnancy test.

A Significant History Point	Test Type and/or Test Technique	Interpretation
Ancient Egypt	cereal grains watered with woman’s urine (sprout test)	if the watered grains sprout—the woman is pregnant
From Hippocrates to Gallen	cereal grains watered with woman’s urine (sprout test)	if the watered grains sprout—the woman is pregnant
	onion test	an onion was inserted into the woman’s vagina and left there overnight—if in the morning a woman’s breath smelled of onions, she was not pregnant
	drinking milk (milk test)	if a woman felt sick after drinking milk, she was pregnant
From the Middle Ages through the Seventeenth Century	visual analysis of a woman’s urine	when the urine is clear, light lemon, turning to whitish, with a foamy surface—the woman is pregnant
	urine/milk flotation test	milk floats on the surface of a pregnant woman’s urine
	urine/needle test	a needle inserted into a vial of pregnant woman’s urine turns rust-red or black
	wine/urine mixing test	precipitation—the woman is pregnant
	sweet drink test before bedtime	navel pain in the morning meant pregnancy
	ribbon/urine odor test	if the smell of a ribbon dipped in a woman’s urine made a woman vomit or feel sick, she was pregnant
	ribbon-urinary-flame test	if the smell of a ribbon dipped in a woman’s urine and burned in a flame makes a woman sick, the woman is pregnant
Nineteenth Century	urine film formation test “Kyesteine pellicle”	if a sticky film forms on the surface of the urine—the woman is pregnant
From the 1920s to the 1960s	Aschheim–Zondek test (mouse/rat test) (laboratory)	a woman’s urine was injected into male rats or female mice—if the rat was sexually aroused and the mice were ovulating, the woman whose urine was injected into the animal was pregnant
	Friedman test (rabbit test) (laboratory)	a woman’s urine was injected into a female rabbit—if the rabbit ovulated, the woman whose urine was injected into the animal was pregnant
	Hogben test (frog test) (laboratory)	a female *Xenopus laevis* frog stimulated by an injection of pregnant woman’s urine produces a spawn
	Galli Maininitest (toad test) (laboratory)	injecting a pregnant woman’s urine into the toad’s (*Bufoarenarum Hensel*) lymphatic sac stimulates the animal to produce sperm
The 1960s	First immunological tests	hemagglutination inhibition test (Wide–Gemzell test)
		latex agglutination inhibition test (slide test)
		complement fixation test
The 1970s	radioimmunoassay (laboratory)	immunological tests using radiation-generating tracers
From the 1970s/1980s to the present	immunochemical tests (laboratory)	enzyme immunoassays using colored or fluorescent markers
		immunochromatographic tests using colored or fluorescent markers
	home pregnancy tests (based on immunological techniques)	complement fixation test
		strip or cassette immunochromatographic tests with colorimetric detection or detection based on biosensors

## Data Availability

Not applicable.
